# Prenatal Lead Exposure and Schizophrenia: A Plausible Neurobiologic Connection

**DOI:** 10.1289/ehp.112-a724a

**Published:** 2004-09

**Authors:** Tomás R. Guilarte

**Affiliations:** Department of Environmental Health Sciences, The Johns Hopkins University, Bloomberg School of Public Health, Baltimore, Maryland E-mail: tguilart@jhsph.edu

In their article in the April issue of *EHP*, Opler et al. (2004) raise the intriguing possibility that prenatal exposure to the ubiquitous developmental neurotoxicant lead (Pb^2+^) may be associated with schizophrenia, an adult psychiatric disease. Although the study has certain limitations that the authors discussed, it brings to light the possibility that prenatal Pb^2+^ exposure may be a risk factor for the expression of schizophrenia later in life. If an association between developmental Pb^2+^ exposure and schizophrenia exists, then identifying plausible neurobiologic substrate(s) would be useful in future studies. A common and potentially critical link between developmental Pb^2+^ exposure and schizophrenia is the disruption of glutamatergic synaptic activity—specifically, hypoactivity of the *N*-methyl-d-aspartate subtype (NMDAR) of glutamatergic receptors.

The “glutamatergic hypothesis” of schizophrenia originated from observations that administration of NMDAR noncompetitive antagonists exacerbates psychotic symptoms in schizophrenics and mimics schizophrenia in nonpsychotic subjects (Coyle et al. 2003; Konradi and Heckers 2003). Further, the administration of such antagonists in animals models certain aspects of the disease. There is experimental evidence that Pb^2+^ is a potent and selective inhibitor of the NMDAR, and the NMDAR plays an important role in neuronal development, synaptic plasticity, and learning and memory (Nihei and Guilarte 2001). Similar to rats exposed to Pb^2+^ during development, several lines of evidence have implicated NMDAR hypofunction in the pathophysiology of schizophrenia (Coyle et al. 2003; Konradi and Heckers 2003).

Developmental exposure to Pb^2+^, in the same concentration range as implied in the work by Opler et al. (2004), alters gene and protein expression of NMDAR subunits in the rat brain (Nihei and Guilarte 2001). A consistent change in NMDAR subunits measured in young adult Pb^2+^-exposed rats is a decrease in NR1 subunit gene expression (Nihei and Guilarte 2001). These findings resemble some of the changes in NMDAR subunit expression described in the brain of schizophrenic patients (Konradi and Heckers 2003; Tsai and Coyle 2002). Further, there is compelling evidence for a common molecular target, the glycine modulatory site of the NMDAR. A proposed mechanism by which Pb^2+^ inhibits NMDAR function is by binding to a divalent cation site associated with the glycine site and allosterically inhibiting glycine binding (Hashemzadeh-Gargari and Guilarte 1999). The significance of the antagonistic action of Pb^2+^ at the glycine site of the NMDAR is that studies have identified abnormalities associated with schizophrenia that interfere with the activation of the glycine modulatory site of the NMDAR (Coyle and Tsai 2004a). Further, the use of NMDAR glycine site agonists such as glycine, d-serine, or d-cycloserine in clinical trials has demonstrated some efficacy in ameliorating the negative symptoms and cognitive disabilities in schizophrenics (Coyle and Tsai 2004a, 2004b).

Although an environmental component to the etiology of schizophrenia has been proposed (Tsuang 2000), developmental Pb^2+^ exposure has not been considered a potential risk factor for schizophrenia until the article by Opler et al. (2004) was published. It is possible that in susceptible individuals, the presence of Pb^2+^ during the development of the central nervous system may be directly related or may contribute to the expression of schizophrenia later in life.
